# A Global Survey on Changes in the Supply, Price, and Use of Illicit Drugs and Alcohol, and Related Complications During the 2020 COVID-19 Pandemic

**DOI:** 10.3389/fpsyt.2021.646206

**Published:** 2021-08-06

**Authors:** Ali Farhoudian, Seyed Ramin Radfar, Hossein Mohaddes Ardabili, Parnian Rafei, Mohsen Ebrahimi, Arash Khojasteh Zonoozi, Cornelis A. J. De Jong, Mehrnoosh Vahidi, Masud Yunesian, Christos Kouimtsidis, Shalini Arunogiri, Helena Hansen, Kathleen T. Brady, ISAM Global Survey Consortium (ISAM-GSC), Adrian Octavian Abagiu, Marc N. Potenza, Alexander Mario Baldacchino, Hamed Ekhtiari

**Affiliations:** ^1^Department of Psychiatry, Tehran University of Medical Sciences, Tehran, Iran; ^2^Department of Neuroscience and Addiction, School of Advanced Technologies in Medicine (SATiM), Tehran University of Medical Sciences, Tehran, Iran; ^3^Integrated Substance Abuse Programs Department, University of California, Los Angeles, Los Angeles, CA, United States; ^4^Psychiatry and Behavioral Sciences Research Center, Faculty of Medicine, Ibn-e-Sina Hospital, Mashhad University of Medical Sciences, Mashhad, Iran; ^5^Student Research Committee, Faculty of Medicine, Mashhad University of Medical Sciences, Mashhad, Iran; ^6^Department of Psychology, Faculty of Psychology and Education, University of Tehran, Tehran, Iran; ^7^Iranian National Center for Addiction Studies, Tehran University of Medical Sciences, Tehran, Iran; ^8^Behavioral Science Institute, Radboud University, Nijmegen, Netherlands; ^9^School of Public Health, Tehran University of Medical Sciences, Tehran, Iran; ^10^Surrey and Borders Partnership National Health Service Foundation Trust, Leatherhead, United Kingdom; ^11^Turning Point, Eastern Health, Box Hill, VIC, Australia; ^12^Departments of Anthropology and Psychiatry, New York University, New York, NY, United States; ^13^Department of Psychiatry and Behavioral Sciences, Medical University of South Carolina, Charleston, SC, United States; ^14^Yale School of Medicine, Connecticut Council on Problem Gambling and Connecticut Mental Health Center, New Haven, CT, United States; ^15^Division of Population and Behavior Sciences, Medical School, University of St Andrews, St Andrews, United Kingdom; ^16^Laureate Institute for Brain Research, Tulsa, OK, United States

**Keywords:** COVID-19, addiction, substance use disorder, global survey, behavioral addiction, illicit drug market

## Abstract

**Background and Aims:** COVID-19 has infected more than 77 million people worldwide and impacted the lives of many more, with a particularly devastating impact on vulnerable populations, including people with substance use disorders (SUDs). Quarantines, travel bans, regulatory changes, social distancing, and “lockdown” measures have affected drug and alcohol supply chains and subsequently their availability, price, and use patterns, with possible downstream effects on presentations of SUDs and demand for treatment. Given the lack of multicentric epidemiologic studies, we conducted a rapid global survey within the International Society of Addiction Medicine (ISAM) network in order to understand the status of substance-use patterns during the current pandemic.

**Design:** Cross-sectional survey.

**Setting:** Worldwide.

**Participants:** Starting on April 4, 2020 during a 5-week period, the survey received 185 responses from 77 countries.

**Measurements:** To assess addiction medicine professionals' perceived changes in drug and alcohol supply, price, use pattern, and related complications during the COVID-19 pandemic.

**Findings:** Participants reported (among who answered “decreased” or “increased”) a decrease in drug supply (69.0%) and at the same time an increase in price (95.3%) globally. With respect to changes in use patterns, an increase in alcohol (71.7%), cannabis (63.0%), prescription opioids (70.9%), and sedative/hypnotics (84.6%) use was reported, while the use of amphetamines (59.7%), cocaine (67.5%), and opiates (58.2%) was reported to decrease overall.

**Conclusions:** The global report on changes in the availability, use patterns, and complications of alcohol and drugs during the COVID-19 pandemic should be considered in making new policies and in developing mitigating measures and guidelines during the current pandemic (and probable future ones) in order to minimize risks to people with SUD.

## Introduction

As of December 23, 2020, the COVID-19 pandemic has around 77 million cases of infection in more than 200 countries with above 1,711,000 overall deaths (1). Approximately 6 months after cases were first diagnosed, there remain few reliable treatments and no vaccines available, and an increasing number of countries are experiencing dangerous COVID-19 transmission (2, 3). Among vulnerable populations to infection and its complications are people with substance use disorders (SUDs) (4). Both comorbid medical conditions in SUDs (such as cardiopulmonary diseases and related risk factors) and drug–drug interactions (between COVID-19 medications and abused substances or SUD treatment medications), along with other factors, may lead to people with SUDs experiencing more complications when encountering COVID-19 infections (4–6).

People with SUDs are vulnerable given marginalization, stigmatization, and poor access to health and social services (7, 8). According to risky behaviors and disadvantaged environments associated with SUDs, people with SUD may not only bear additional risks for COVID-19 but also experience poorer outcomes (4). Therefore, during the pandemic, gathering current information on the status of SUD is critical to support planning and mobilizing timely responses to minimize risks (4). Alterations in alcohol and drug supplies may change prices and availability and therefore use patterns. The COVID-19 pandemic has resulted in quarantines, travel bans, regulatory changes, and social distancing “lockdown” measures globally, with impacts on supply chains. In the setting of COVID-19-related stressors, there may be decreases in drug and alcohol availability, increases in price and use patterns, and possible downstream effects on SUD presentations and treatment demands. Such changes could directly/indirectly affect people with SUDs and give rise to new challenges and additional needs in the field of addiction medicine. Drug shortages, as the United Nation Office for Drug and Crime (UNODC) reports, could have negative health consequences regarding transitioning to consumption of harmful domestically produced substances along with more dangerous patterns of drug use including shifting to injections and using shared drug administration equipment, especially in the case of heroin (9). Additionally, the lack of drug supply may result in higher prices for some substances and bring financial burden to drug users and increase the odds of risky/illegal behaviors (4). Concurrently, as legal liquor shops may remain closed during the lockdown in some countries, multiple problems may occur ranging from alcohol withdrawal to toxicity and death due to shifting to low-quality homemade liquor and accidental methanol ingestion (4, 10).

People with SUDs could be exposed to some indirect risks during the COVID-19 era as well (5). For instance, as healthcare facilities become more difficult to access during lockdowns, people with SUDs may experience more difficulties relating to poor access to treatment centers. Socioeconomically disadvantaged backgrounds and diminished availability of public transportation may exacerbate such concerns (4, 5, 11), especially for individuals receiving daily prescriptions of opioid substitution therapy (4). Professional authorities and health policymakers are expected to proactively address such emerging needs. However, the lack of reliable data complicates the generation and implementation of evidence-based policies.

Although some activities and reports from different worldwide organizations have initially responded to the COVID-19 pandemic, data provided have been limited and, in some occasions, as UNODC has reported, the information base for analyses has been restricted and feasibility of implementation unknown^1^ (12– 16). Thus, a vacancy exists for a comprehensive report describing the global situation with respect to drug use, drug supply, and related complications.

In order to formulate a comprehensive health response, it is important to understand alcohol and drug markets' situation (availability and price), use patterns and related complications, and how they may have changed during the pandemic. Designing a global in-depth epidemiologic study, apart from questions about its feasibility, is challenging during the pandemic. Therefore, the International Society of Addiction Medicine (ISAM) designed a comprehensive global survey and collected expert opinions on perceived changes in substance use situation and health system responses around the 1st week of April 2020 in what aims to be a longitudinal study (17).

Here, we report results from the first round of the ISAM global survey on drug and alcohol use, price, supply, and complications during the COVID-19 pandemic. Data related to the second section of the survey concerning substance use treatment and harm reduction services responses to the pandemic have been published recently (18). We hypothesized that drug and alcohol use would increase, prices would increase, supply would decrease, and complications would increase and that results would differ by region (given the differential spread of COVID-19 and regional responses to the COVID-19 pandemic). We hope that current data will help to address the urgent need for more accurate information about the status of drug and alcohol use in the current pandemic and provide information about appropriate modifications in health system services to respond to the emerging demands in the current pandemic and similar potential pandemics in the future.

## Methods

### Sample

The complete study protocol has been previously published (17). The ISAM mailing list (and subsequent snowballing methodologies) comprising addiction medicine professionals across the world were contacted on April 4, 2020 by email with an invitation to participate in the study by clicking on a link to the online survey. The invitees were informed that the survey will ask about their opinions and information toward COVID-19 pandemic impact on SUDs. They also initially consented to be included as an author in the publications following the survey. Those who approved the manuscript and authorship were included among the main authors or the ISAM Global Survey Consortium (ISAM-GSC) based on their contribution in this project. Data collection was concluded on May 8, 2020.

### Questionnaire

The questionnaire consisted of 92 questions in two main sections: (1) situational assessment during the pandemic and (2) health response to the pandemic. This paper provides an analysis of data obtained from the situation assessment section of the survey concerning changes in drug use, supply, price, risky behaviors, as well as related measures, namely morbidities, mortalities, and overdose rates during the COVID-19 pandemic period in different countries (17). Questions on the situational assessment section of the survey are available in Supplementary Method 2. The questionnaire was distributed in English for all the respondents.

### Statistical Analysis

All statistical analyses were conducted using RStudio (v. 1.2.1335). Descriptive data are presented as means and percentages for each country's response, as well as the average of the global responses.

### Ethics Approval

The survey protocols and all materials, including the survey questionnaires, received approval from the University of Social Welfare and Rehabilitation Sciences', ethics committee in Tehran, Iran (Code: IR.USWR.REC.1399.061).

## Results

### Respondents' Global Distribution

**Figure 1:**
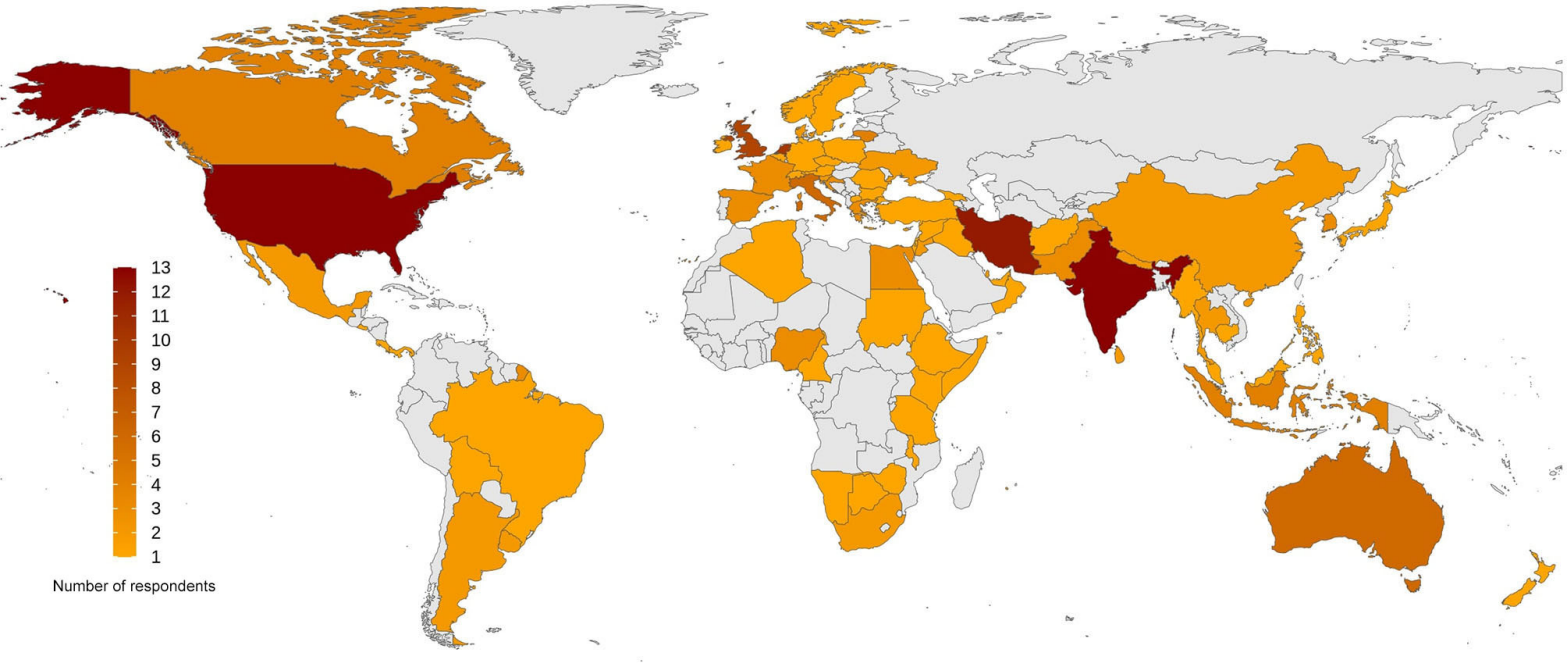
Global distribution of the respondents to the survey. The survey involves 177 respondents from 77 countries around the world, ranging from 1 to 13 participants from each country, demonstrated as a color spectrum from orange to dark red.

Overall, 185 respondents from 77 countries participated. Eight responses were excluded because of insufficient information provided (the “insufficient information” was predetermined as having more than 50% of “I do not know” responses). Data from the rest of the 177 respondents were analyzed. The list of the countries that provided information for this survey is available as a supplement (Supplementary Method 1). Figure 1 depicts a map of the respondents' global distribution.

### Respondents' Demographic Characteristics

Respondents consisted of 111 males (62.7%), 62 females (35%), and 4 people (2.3%) who selected “other” or preferred not to disclose their gender. The mean age of the respondents was 46.51 ± 10.78 years. Most respondents were medical professionals (MDs) (*n* = 148, 83.6%), and the most frequent primary discipline was psychiatry (*n* = 95, 53.7%). Information related to the respondents' main disciplines and academic degrees is shown (Table 1).

**Table 1:** The demographic and professional information of survey respondents including their gender, age, academic degree, and primary discipline.

		**N/Mean (± SD)**	**Percent (%)**
**Gender**			
	Male	111	62.7
	Female	62	35
	Other/not disclosed	4	2.3
**Age in years**		46.51 (± 10.78)	
**Academic qualification/s**			
	BSc	6	3.4
	MSc	13	7.3
	MD	72	40.7
	MD; MSc	13	7.3
	MD; PhD	32	18.1
	PhD	31	17.5
	Others	10	5.6
**Primary professional discipline**			
	Addiction medicine	19	10.7
	Drug/health policy	8	4.5
	General medicine	17	9.6
	Pharmacology	2	1.1
	Psychiatry	95	53.7
	Psychology/counseling	20	11.3
	Social work	5	2.8
	Other medical specialties	3	1.7
	Others	8	4.5

### Drug use During Pandemic

**Figure 2:**
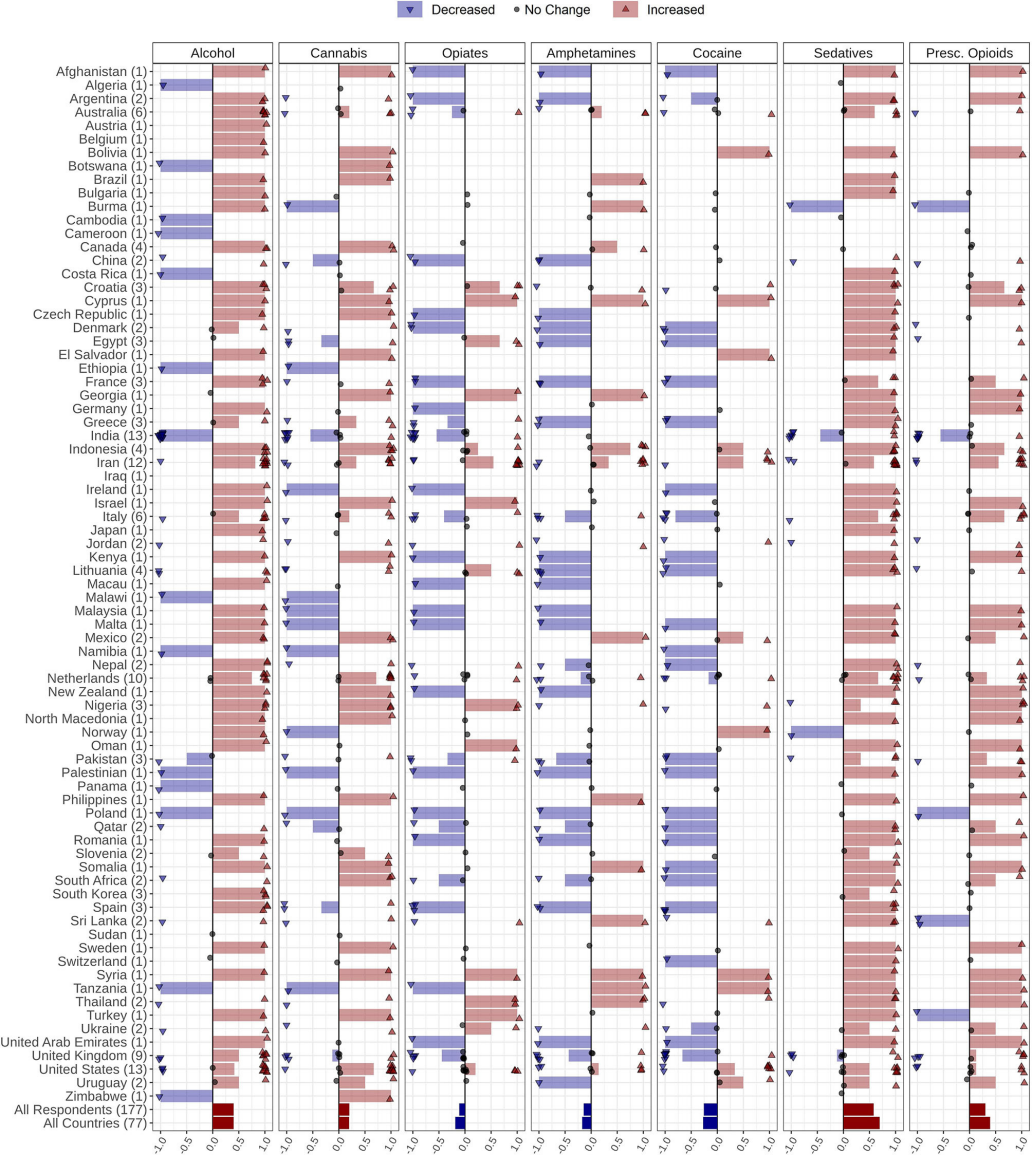
Changes in alcohol and drug use during the COVID-19 pandemic reported by 177 respondents from 77 countries globally. Respondents were asked to report changes in alcohol, amphetamines, cannabis, cocaine, opiates, prescribed opioids, and sedative-hypnotics use with the following options: *Increased, Decreased, Not changed, I do not know*, and *Number of users is very low/none*. Countries' names are sorted in alphabetical order, and the number of respondents from each country is in parentheses following the country name. Each response is indicated as a single dot for *no change* or up and down triangles for *increased* and *decreased* answers, respectively, with a minor jitter for better visualization. The reported answers are represented as −1 for *decreased*, 1 for *increased*, and 0 for *no change. I do not know* and *Number of users is very low/none* answers are not shown in the figure. The mean of all responses, regardless of their originated countries and without considering those who did not know the answer or reported very low/none number of users, alongside the average answers of all countries, regardless of the number of respondents in each country, are addressed in the last two rows below the countries' names (Pres. Opioids: prescription opioids).

Respondents provided information about drug use changes in their countries during the COVID-19 pandemic. Over 63% (*n* = 49), 42% (*n* = 32), 64% (*n* = 50), and 41% (*n* = 32) of the countries reported that use of alcohol, cannabis, sedatives, and prescription opioids increased, respectively. Conversely, opiates, amphetamine, and cocaine use has seen a decrement in 31% (*n* = 24), 29% (*n* = 22), and 29% (*n* = 23) of the countries, respectively. Perceived drug use changes by country are shown (Figure 2, Table 2). Details of drug use changes are reported in Supplementary Material.

**Table 2:** Summary of the survey responses in different sections related to situational assessment including respondents' information about changes in alcohol and drug use pattern, supply, price, morbidity and mortality, and overdose.

	**Responders (177)**								**Countries (77)**							
	**Decrease**		**No change**		**Increase**		**Others**		**Decrease**		**No change**		**Increase**		**Others**	
	***N***	**%**	***N***	**%**	***N***	**%**	***N***	**%**	***N***	**%**	***N***	**%**	***N***	**%**	***N***	**%**
**Use**																
Alcohol	43	24%	13	7%	**109**	**62%**	12	7%	19	25%	6	8%	**49**	**63%**	3	4%
Cannabis	44	25%	35	20%	**75**	**42%**	23	13%	20	26%	17	22%	**32**	**42%**	8	10%
Opiates	**53**	**30%**	43	24%	38	21%	43	24%	**24**	**31%**	16	20%	14	18%	23	30%
Amphetamines	**49**	**28%**	30	17%	33	19%	65	37%	**22**	**29%**	15	20%	14	18%	26	33%
Cocaine	**52**	**29%**	28	16%	25	14%	72	41%	**23**	**29%**	15	19%	10	14%	29	38%
Sedatives	19	11%	24	14%	**105**	**59%**	29	16%	5	6%	9	11%	**50**	**64%**	14	18%
Presc. Opioids	27	15%	35	20%	**66**	**37%**	49	28%	8	11%	16	21%	**32**	**41%**	21	27%
**Supply**																
Alcohol	**62**	**35%**	49	28%	52	29%	14	8%	**26**	**34%**	21	28%	24	31%	6	7%
Cannabis	**62**	**35%**	46	26%	33	19%	36	20%	**29**	**37%**	18	24%	15	20%	15	19%
Opiates	**71**	**40%**	34	19%	18	10%	54	31%	**31**	**41%**	14	18%	6	8%	26	33%
Amphetamines	**54**	**31%**	34	19%	19	11%	70	40%	**29**	**38%**	14	18%	7	9%	27	35%
Cocaine	**56**	**32%**	32	18%	15	8%	74	42%	**26**	**34%**	14	18%	7	9%	30	39%
**Price**																
Alcohol	5	3%	**91**	**51%**	57	32%	24	14%	3	4%	**42**	**54%**	23	29%	10	13%
Cannabis	4	2%	51	29%	**70**	**40%**	52	29%	2	3%	23	30%	**30**	**39%**	22	28%
Opiates	2	1%	33	19%	**75**	**42%**	67	38%	2	2%	14	18%	**29**	**37%**	33	43%
Amphetamines	3	2%	33	19%	**57**	**32%**	84	47%	2	3%	13	17%	**26**	**34%**	35	46%
Cocaine	1	1%	35	20%	**51**	**29%**	90	51%	0	0%	18	23%	**21**	**28%**	37	49%
**Morbidity and mortality**																
Alcohol	7	4%	34	19%	**72**	**41%**	64	36%	5	7%	16	21%	**27**	**35%**	29	38%
Drug	7	4%	34	19%	**68**	**38%**	68	38%	5	6%	14	18%	**28**	**36%**	30	39%
**Overdose**	14	8%	**53**	**30%**	35	20%	75	42%	7	9%	**24**	**32%**	14	18%	32	42%

*The mean number and percentage of Increased, Decreased, No Change, and “Others” responses, regardless of their originated countries and the average answers of all countries, regardless of the number of respondents in each country. “Others” indicate responses that involved respondents' lack of information or reluctance for responding to the relevant question. The bold values indicates highest rates of responses among respondents and countries*.

Respondents were also asked to report changes in behavioral addictions (gaming/gambling) in their countries through the following options: Increased, Decreased, No change, I do not know; 85.7% (*n* = 66) of the countries reported that behavioral addictions rates had increased, whereas 14% (*n* = 11) of the countries reported that behavioral addictions rates had decreased in their countries during the COVID-19 pandemic (Supplementary Figure 1).

### Drug Supply

Respondents provided information about perceived drug supply changes in their countries during the COVID-19 pandemic. The drug categories included the following: alcoholic beverages, cannabis (including marijuana and synthetic cannabinoids such as spice, K2, etc.), opiates (including opium, heroin, opium residue, etc.), amphetamine-type stimulants (including amphetamine, methamphetamine, MDMA, etc.), and cocaine (including crack cocaine).

**Figure 3:**
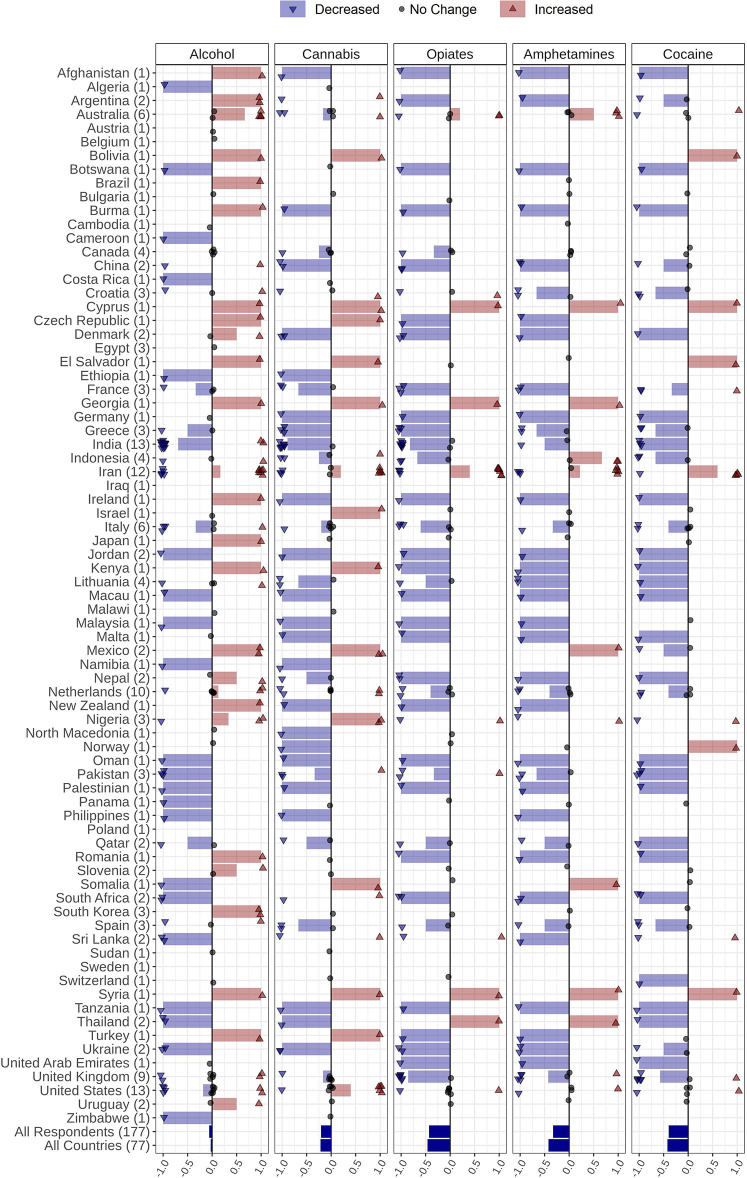
Changes in alcohol and drug supply during the COVID-19 pandemic reported by 177 respondents from 77 countries globally. Respondents were asked to report changes the supply of alcohol, amphetamines, cannabis, cocaine, and opiates through the following options: *Increased supply, decreased supply, no change*, and *I do not know*. Countries' names are sorted in alphabetical order, and the number of respondents from each country is in parentheses following the country name. Each response is indicated as a single dot for *no change* or up and down triangles for *increased* and *decreased* answers, respectively, with a minor jitter for better visualization. The reported answers are represented as −1 for *decreased*, 1 for *increased*, and 0 for *no change; I do not know* answers are not shown. The mean of all responses, regardless of their originated countries and without considering those who did not know the answer, alongside the average answers of all countries, regardless of the number of respondents in each country, are addressed in the last two rows below the countries' names.

Decreased supply patterns for all substances were noted. A decrement was reported in supply in 34% (*n* = 26) of the countries for alcohol, 37% (*n* = 29) for cannabis, 41% (*n* = 31) for opiates, 38% (*n* = 29) for amphetamines, and 24% (*n* = 26) for cocaine (Figure 3, Table 2). Details of drug supply changes are reported in the Supplementary Material.

### Drug Price

**Figure 4:**
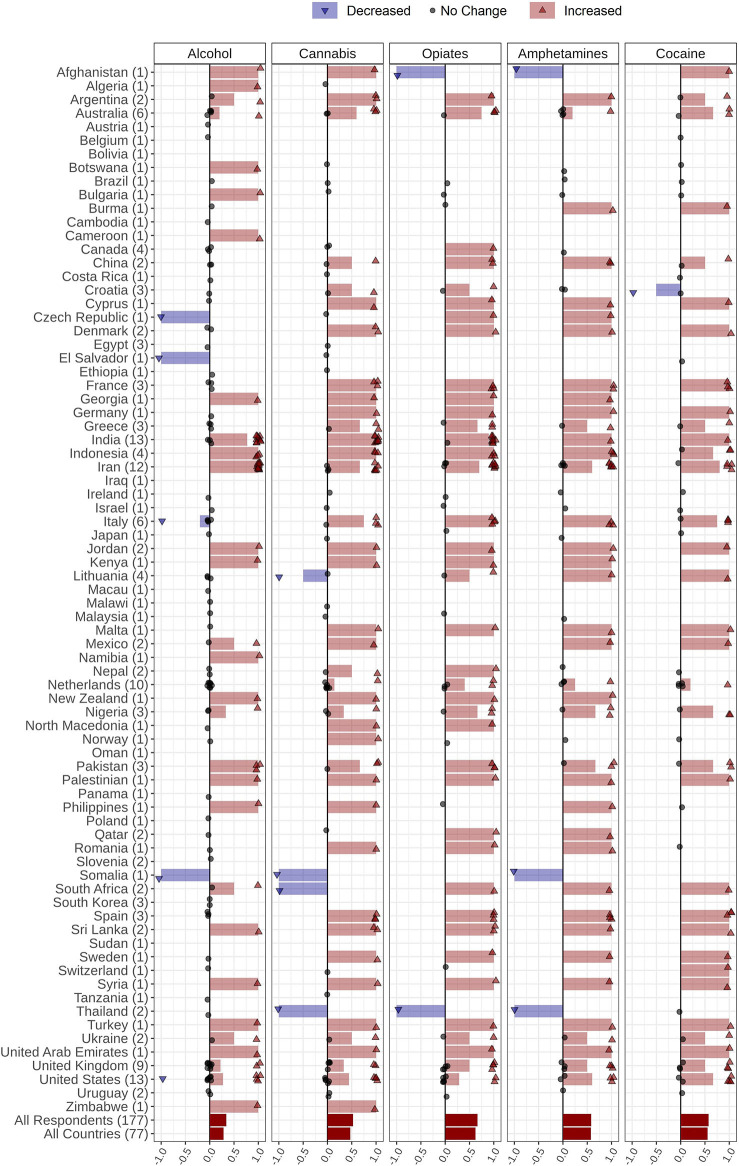
Changes in alcohol and drug prices during the COVID-19 pandemic reported by 177 respondents from 77 countries globally. Respondents were asked to report changes in alcohol, amphetamines, cannabis, and opiates prices through the following options: *Price increased, Price decreased, Price did not change*, and *I do not know*. Countries' names are sorted in alphabetic order, and the number of respondents from each country is in parentheses following the country name. Each response is indicated as a single dot for *no change* or up and down triangles for *increased* and *decreased* answers, respectively, with a minor jitter for better visualization. Reported answers are represented as −1 for *decreased*, 1 for *increased*, and 0 for *no change; I do not know* answers are not shown in the figure. The mean of all responses, regardless of their originated countries and without considering those who did not know the answer, alongside the average answers of all countries, regardless of the number of respondents in each country, are addressed in the last two rows below the countries' names.

Respondents provided information regarding perceived drug price changes in their countries during the COVID-19 pandemic. The price of cannabis, opiates, amphetamines, and cocaine increased in 39% (*n* = 30), 37% (*n* = 29), 34% (*n* = 26), and 28% (*n* = 21) of the countries, respectively. Alcohol price was reported as unchanged in 54% (*n* = 42) of the countries (Figure 4, Table 2). Details of drug price changes are reported in the Supplementary Material.

The information related to changes in drug price among different countries is shown in Figure 4 and Table 2.

### Perceived Morbidity and Mortality (Including Overdose)

**Figure 5:**
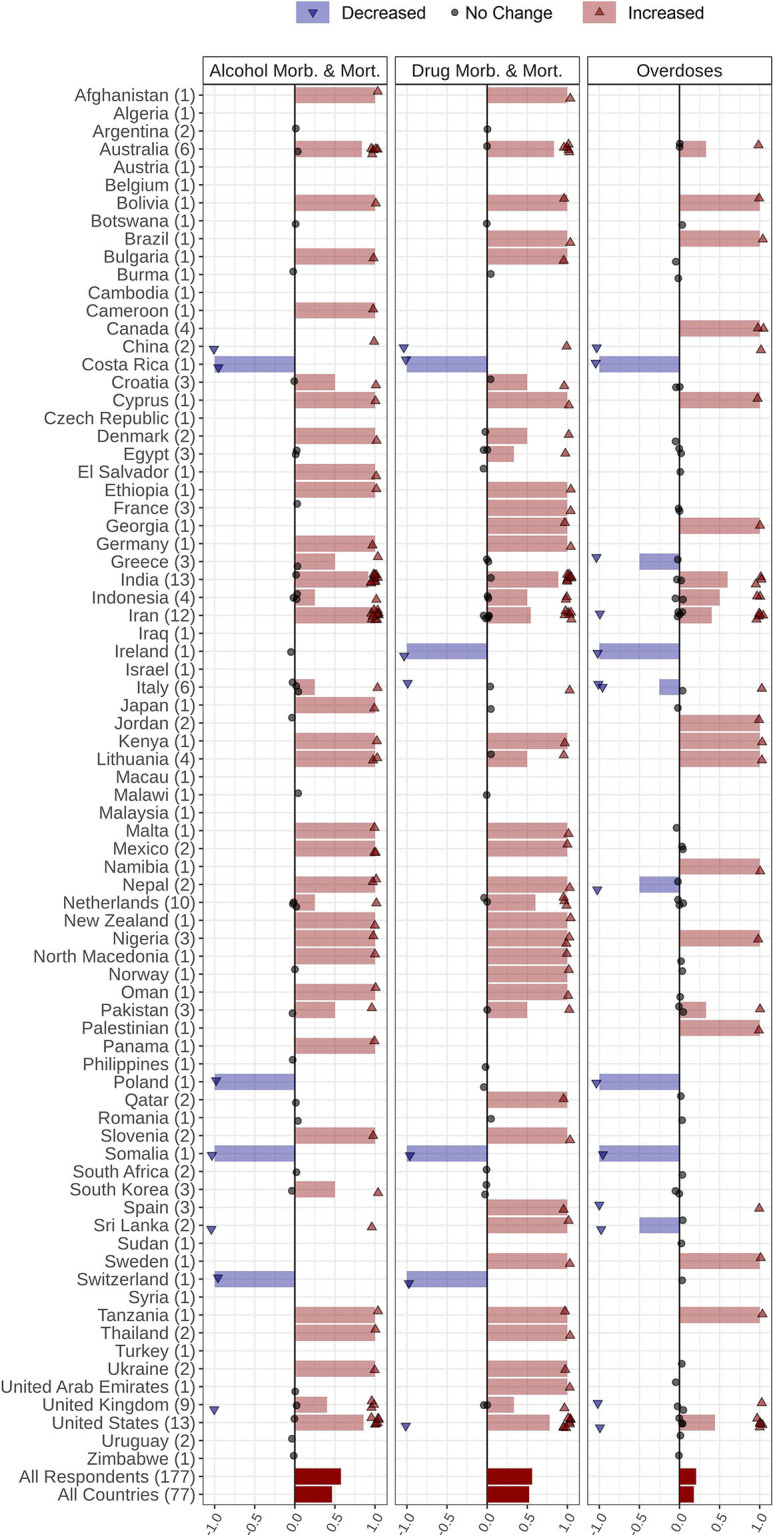
Changes in mortality, morbidity, and overdose in people with SUD during the COVID-19 pandemic reported by 177 respondents from 77 countries around the world. Respondents were asked to report changes in morbidity or mortality rates in people with SUD and changes in fatal and non-fatal overdose episodes through the following options: *Increased, Decreased, I do not know, I do not like to answer*, and *Not applicable*. Countries' names are sorted in alphabetical order, and the number of each country's respondents is mentioned in front of the names. Each response is indicated as a single dot for *no change* or up and down triangles for *increased* and *decreased* answers, respectively, with a minor jitter for better visualization. The reported answers are represented as −1 for *decreased*, 1 for *increased*, and 0 for *no change; I do not know, I do not like to answer*, and *Not applicable* answers are not shown in the figure. The mean of all responses, regardless of their originated countries and without considering those who did not know the answer, alongside the average answers of all countries, regardless of the number of respondents in each country, are addressed in the last two rows below the countries' names (SUD, Substance Use Disorder).

Respondents provided information about whether morbidity and mortality, including fatal and non-fatal overdose rates, in their countries had changed during the COVID-19 pandemic. Mortality rates in people with alcohol use disorders (AUDs) and SUDs were reported to have increased in 35% (*n* = 27) and 36% (*n* = 28) of the countries, respectively. No changes in fatal and non-fatal overdose rates were reported by 32% (*n* = 24) of the countries (Figure 5, Table 2). Details of changes in mortalities and overdose rates are reported in the Supplementary Material.

### Risky Behaviors

**Figure 6:**
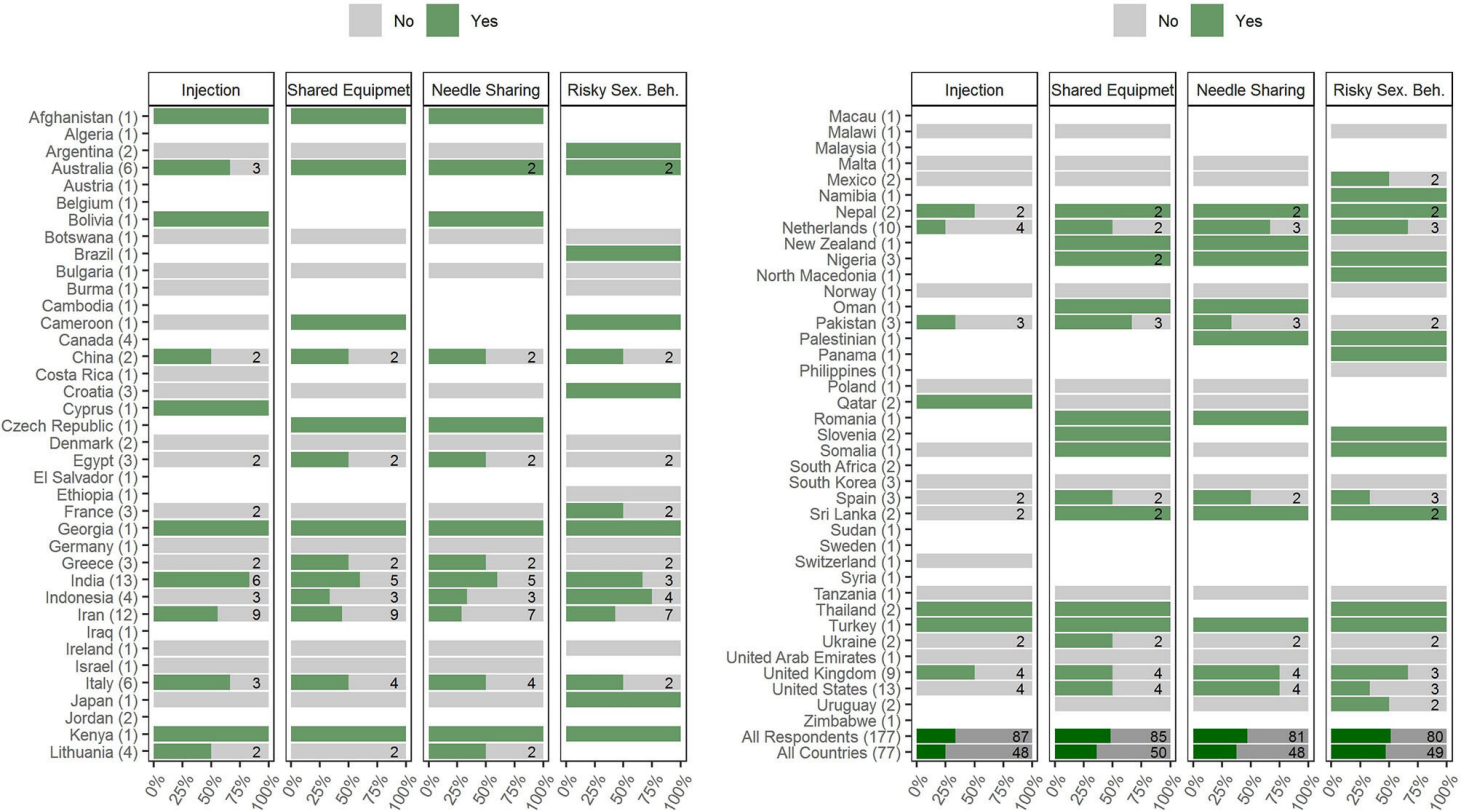
Changes in risky behaviors including shifting to injection, using shared drug use equipment, needle sharing, and risky sexual behaviors during the COVID-19 pandemic period, reported by 177 respondents from 77 countries globally. Respondents were asked to report changes in risky behaviors (injection, shared drug use equipment, needle sharing, and risky sexual behaviors) through the following options: *Yes, No, I do not know, I do not like to answer*, and *Not applicable*. Countries' names are sorted in alphabetical order, and the number of each countries' respondents is mentioned in front of the names. The numbers of respondents who reported *Yes* or *No* answers to each question are demonstrated inside the bars (If nothing is written, it indicates that there was only one response within *Yes* and *No* answers). The percentages shown by the bars are also based on only *Yes* or *No* answers. The mean percentages of all responses, regardless of their originated countries and without considering those who reported other than *Yes* and *No* answers, alongside the mean percentage answers of all countries, regardless of the number of respondents in each country, are addressed in the last two rows below the countries' names (Risky Sex. Beh., Risky Sexual Behaviors).

Respondents provided information about changes in risky behaviors among people with SUDs in their countries during the COVID-19 pandemic (Figure 6, Supplementary Table 1). Information related to risky behaviors consisted of increased/switching to injection, sharing drug use equipment, needle and syringe sharing, and risky sexual behaviors. Sixteen percent (*n* = 29) of the respondents reported that injection among people with SUDs has increased, while 33% (*n* = 58) reported no change in numbers of people injecting drugs or people switching to injection. Fifty-one percent (*n* = 90) chose the “others” option indicating a lack of information or reluctance in responding to this question. Twenty-three percent (*n* = 41) of the respondents reported that sharing drug use equipment (i.e., paraphernalia) has increased, while 25% (*n* = 44) reported no change. Fifty-two percent (*n* = 92) chose the “others” option indicating a lack of information or reluctance in responding. Twenty-one percent (*n* = 38) reported that sharing needle and syringe has increased, while 24% (*n* = 43) reported no change. Fifty-four percent (*n* = 96) chose the “others” option indicating a lack of information or reluctance in responding to this question. Twenty-three percent (*n* = 41) reported that risky sexual behaviors have increased, while 22% (*n* = 39) reported no change. Fifty-five percent (*n* = 97) chose the “others” option. Respondents reported an increase in behavioral addictions during the pandemic (Supplementary Figure 1).

### COVID-19 Overall Impact on SUDs

**Figure 7:**
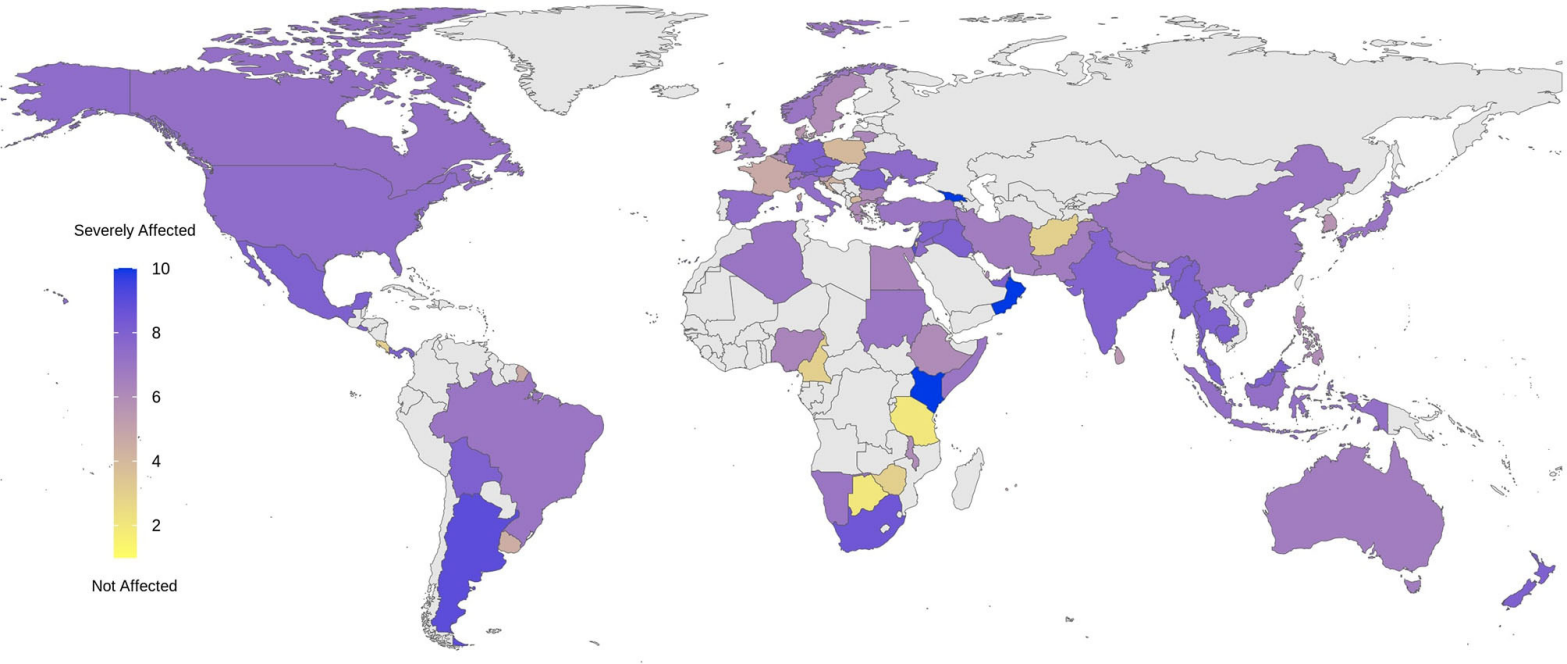
Severity of being affected by COVID-19 outbreak among people with SUDs reported by 177 respondents from 77 countries. Addiction medicine professionals were asked to report how seriously people with SUDs in their countries have been affected by the COVID-19 pandemic using a range of between 1 and 10: 1 representing *Not affected*, demonstrated with yellow at the beginning of the spectrum, and 10 representing *Severely affected* at the end of the spectrum, indicated with blue. Responses were collected beginning April 4, 2020 and through a 5-week period.

Respondents provided an overall rating of the general impact of the COVID-19 pandemic on people with SUDs in their countries (Figure 7). Respondents from Oman, Kenya, and Georgia rated the highest severity of COVID-19 impact on people with SUDs in their countries (ratings of 10/10), while respondents from Botswana and Afghanistan rated the lowest severity for this impact in their countries (ratings of 2/10).

## Discussion

According to the results of this first-ever COVID-19 and SUD global survey with the contribution of 177 addiction medicine professionals/policymakers from 77 countries, the majority of respondents believed that in their countries, people with SUDs had been seriously affected by the COVID-19 outbreak. They mostly believed that prices for alcohol and drugs have risen, and they have become less available during the pandemic. In regard with alterations in use patterns, respondents perceived an increase the use of alcohol, cannabis, prescribed opioids, and sedative/hypnotics, and a decrease in the use of amphetamines, cocaine, and opiates. Most respondents reported increases in complications related to drug and alcohol use including increased morbidity and mortality in people with SUDs.

Alterations in levels of alcohol consumption during pandemic are similar to those reported during prior social crises, like the 2008–2009 economic downturn (19). Changes in alcohol consumption may arise from two potentially contradictory, however interacting mechanisms: (1) a problematic increase, usually stemming from distress that is being experienced especially at the beginning of a crisis, or in an attempt to “stockpile”; or (2) a decrease due to the lack of access and financial difficulties, which may lead to withdrawal (20). Current reports from Australia indicate increases in purchases of alcoholic beverages during lockdown potentially due to the first mechanism (21). However, India seems to be encountering a surge in numbers of individuals withdrawing from alcohol (5, 22). These independent reports from Australia and India are in line with our survey findings (Figure 2). Initial reports from Australia and the United States indicate overall increases in alcohol sales, especially in online alcohol delivery subsectors (21), although specific data from the industry on alcohol supply are largely lacking. However, there was no consensus among our survey respondents about changes in alcohol supply, as the responses that reported an increase, decrease, and no change were approximately equal. Approximately half of our survey respondents believed that there is no change in alcohol cost during the pandemic. This is while almost another half reported an increase in alcohol prices. We could not find any relevant reports indicating alcohol price alterations. Further data are needed as the pandemic progresses and hopefully resolves.

There are currently concerns about morbidity and mortality spikes within people with AUDs and alcohol-associated liver disease during the pandemic (23). The survey's results support the idea that these spikes can be seen among people with AUDs. Reports from Iran describe methanol poisoning of around 5,000 people with nearly 700 deaths, which may be due to lack of education and illegal and uncontrolled alcohol sales because of alcohol bans in Iran (10, 24, 25). However, to the best of our knowledge, there are yet no specific reports demonstrating the extent of alcohol overdose. The same pattern also applies to drug-related mortalities and morbidities.

Survey results suggest increases in cannabis use in more than half of participating countries. The European Monitoring Centre for Drugs and Drug Addiction (EMCDDA) has investigated this matter through three large darknet markets (26) in the first 3 months of 2020 and reported overall increased market activity, mostly in relation to cannabis products (13, 27). This might show the initial effects of the pandemic on the European countries market, particularly before peaks in the number of people infected by COVID-19 and subsequent widespread lockdowns.

Opiates, amphetamines, and cocaine were generally reported to have a decrease or no change in patterns of usage in most countries. During the 2008 global financial crisis, drug use patterns were differentially impacted, with expenditures of money for drugs down 2–44%, termed as the “Great Recession” of drug use (19). Although there are preliminary reports suggesting that opioid use is a risk factor for ICU admission in H1N1 infections and a possible risk factor for mortality following COVID-19 infection, rumors about protective effects of opium use in Iran may have led to increased consumption (28, 29). In the US, an already severe opioid overdose crisis worsened since the COVID-19 pandemic, with 30 out of 50 states reporting increases in overdoses between March and June of 2020, with an increase in high potency synthetic opioids such as fentanyl in street supplies and decreased access to harm reduction and OUD treatment services cited as possible drivers of overdose increase (30–32). While concerns have been also raised regarding probable effects of substances on COVID-19 patients (4, 33, 34), more research is needed on changes in drug use patterns and impacts on SUDs.

More than 80% of the countries reported increased use of sedatives and hypnotics. This rise in the demand for sedatives/hypnotics may be related to the stressful situation of the COVID-19 pandemic and its consequences. Survey results also suggest increased use of prescription opioids, perhaps for similar reasons, and changes in services may be needed (35, 36). Canada, Australia, United Kingdom, and Scotland facilitated pharmacy-based methadone-dispensing programs as prescribing opioid-related medications increased (36). This model may help to manage withdrawal syndromes during lockdown-related periods. In the United States, rapid changes in policies provided support to facilitate service delivery for people in opioid treatment programs, such as larger quantities of dispensed methadone and buprenorphine and relaxed regulations around virtual prescriber visits to initiate and continue medications for OUD in order to help patients access and maintain access to medications (35, 37).

The EMCDDA has reported recent increases in the drug demands in European markets (13). The EMCDDA has also noted that due to increases in the retail prices of cannabis and cocaine, the localized supply shortages may exist during the pandemic (12). The UNODC has reported that across all regions globally, many countries have noted a general shortage of different drugs at the retail level, mostly due to reduction in imports or strict lockdown rules, resulting in fewer personal interactions for drug sales (14). The UNODC has also noted a heterogeneous situation on bulk supply, both across drugs and across different countries (14). The UNODC preliminary data were gathered from governmental authorities and open sources (media and UNODC field officers) (14). Our results agree with multiple aspects of these reports of drug supplies.

The UNODC reported that countries with strict rules on social distancing such as the Czech Republic, United Kingdom, Italy, and Iran have been facing increased street drug prices due to lack of availability (14). Other reports from drug-producing countries suggest drug price decrements perhaps as a result of stockpiling of drugs (14). Subsequently, the EMCDDA along with the UNODC have both noted that COVID-19 restrictions have generally led to increases in drug prices, including cocaine, heroin, amphetamines, and cannabis, at the level of street markets (13, 14). Our survey results support these preliminary data reported by the UNODC and EMCDDA.

Respondents mostly reported increases in behavioral addictions during the current pandemic, which may partly confirm the existing concerns on this matter (38, 39). Other small studies suggest increases in addictive behaviors (39–41). Some forms of gambling may have decreased due to financial uncertainties, occupational problems, cessation of sporting events, closure of casinos, and other factors (40, 41). Discussing another addictive behavior, gaming has been represented to be a coping mechanism during the current stressful conditions (42). Accordingly, gaming has increased among college students in India, who use gaming as an antistress mechanism (42). Increased gaming has been occurring globally during the pandemic (43), as well as pornography viewing (44). These and other concerns have led to guidance about Internet use during the pandemic (45).

## Advantages and Limitations

ISAM conducted the first global survey in the field of addiction medicine and successfully sampled responses from 77 countries and 177 experts globally. This timely and rapid survey was designed in a multistep fashion including literature review, expert communication, professional qualitative appraisal, and finally a pilot study (17) and was able to rapidly and reliably address urgent gaps in knowledge during the current pandemic. However, there are limitations such as heterogeneity the numbers of respondents from different countries and their disciplines and educational levels. The convenience sample also may impact response rates and other factors. The lack of validated measures is a limitation, as is the lack of options for open-ended responses that would provide a window on the mechanisms driving reported trends. The fact that not all the countries across the world are included in the study may question the nature of the word “global,” which has been used throughout the survey. Given the dynamic nature of pandemics and lack of multicentric epidemiological studies, the survey is a timely approach to provide a snapshot of global clinical addiction medicine concerns during these unprecedented times.

## Conclusions and Practical Implications

The objective of the ISAM survey was to provide initial, rapid preliminary evidence about how COVID-19 has affected different situational aspects experienced by people with SUDs globally in order to help reach a better understanding of the current status. Provision of this information to international organizations and regional policymakers should help authorities plan for addressing urgent needs and providing suitable services not only in the current pandemic but also in future similar situations. To properly respond to the emerging demands and situational shifts during the COVID-19 pandemic in the addiction treatment services across the world, at a **macro (policy) level**, it is critical to recognize the importance of (1) the social safety net and measures used to reduce the social inequality widening gap when such epidemics deteriorate an already vulnerable system, (2) responsive and publicly well-resourced healthcare with adequate supply of appropriate medication, (3) civil liberties, which could help increased participation and a judicious response by law enforcement agencies, and (4) policies that have taken in justifying alcohol sales and cannabis dispensaries as essential services and legislation allowing pharmacists to provide maintenance medications such as benzodiazepines in order to guarantee safe supplies. At a **meso (organizational) level**, it is important that clinical experience and knowledge on localized drug supply, price, and associated morbidities and mortality is shared within the organization in order to respond adequately. This makes it vital that organizations have a responsive continuity plan that can change with the needs of the population throughout the acute stage of the pandemic. It is also important to establish, support, and sustain varied digital platforms to allow better access to treatment for drug and alcohol using populations and minimize morbidities and possibly mortality. Establishing joint advocacy groups of service users and providers is also critical. At a **micro (individual) level**, it is important to (1) establish a mechanism for shared decision making through effective communication channels, (2) build the therapeutic environment that welcomes and encourages participation of peer, third sector, and/or frontline workers who are also involved in the care of the individuals in care, (3) support psychologically informed environments and interventions considering stress, uncertainties, isolation, and mental health, and (4) consider providing harm minimization and/or public protection messages and equipment to all in care and others.

In this unique global survey, experts in addiction medicine provided information on changes in regional alcohol and drug availability, price, usage, and related complications. Reported decreases in alcohol and drug supplies appear partly attributable to lockdowns, import/export limitations, and strict regulations. Reduced availability may have generated increases in prices. Reported increases in the use of alcohol, cannabis, prescribed opioids, and sedative/hypnotics may reflect their legal availability (in online markets, drugstores, and dispensaries), while decreased use of amphetamines, cocaine, and opiates may be related to decreased availability due to social distancing, lockdown regulations, and increased prices. Changed drug use patterns may not only impact people with SUDs but also give rise to risky behaviors and related complications. Most issues may potentially be preventable if future lockdown regulations are accompanied by enhanced service provision for at-risk communities.

## Data Availability Statement

The raw data supporting the conclusions of this article will be made available by the authors, without undue reservation.

## Ethics Statement

The studies involving human participants were reviewed and approved by the survey protocols and all materials, including the survey questionnaires, received approval from the University of Social Welfare and Rehabilitation Sciences' ethics committee in Tehran, Iran (Code: IR.USWR.REC.1399.061). The participants provided their written informed consent to participate in this study.

## Author Contributions

AF, SR, HE, CD, and AB conceived and designed the study. AF, SR, PR, MV, HE, CD, and AB conducted the survey and collected the data. ME and PR analyzed the data and ran the statistical analyses. AF, SR, HE, CD, MY, and AB supervised the analysis and gave conceptual advice. HM, AZ, PR, and HE contributed to drafting the first draft of the manuscript. AB, MP, SA, and CK edited the manuscript. All authors discussed the results, implications, and commented on the final manuscript.

## International Society of Addiction Medicine—Global Survey Consortium (ISAM-GSC) Members

Adrian Octavian Abagiu^1^, Franck David Noel Abouna^2^, Mohamed Hassan Ahmed^3^, Basma Al-ansari^4^, Feda Mahmmoud Abu Al-khair^5^, Mandhar Humaid Almaqbali^6^, Atul Ambekar^7^, Sidharth Arya^8^, Victor Olufolahan Asebikan^9^, Murad Ali Ayasreh^10^, Debasish Basu^11^, Zoubir Benmebarek^12^, Roshan Bhad^7^, Mario Blaise^13^, Nicolas Bonnet^14^, Jennifer Brasch^15^, Barbara Broers^16^, Anja Busse^17^, Jenna L. Butner^18^, Moses Camilleri^19^, Giovanna Campello^17^, Giuseppe Carra^20^, Ivan Celic^21^, Fatemeh Chalabianloo^22^, Abhishek Chaturvedi^23^, José de Jesús Eduardo Noyola Cherpitel^24^, Kelly J. Clark^25^, Melissa Anne Cyders^26^, Ernesto de Bernardis^27^, Abbas Deilamizade^28^, John Edward Derry^29^, Naveen Kumar Dhagudu^30^, Pavla Dolezalova^31^, Geert Dom^32^, Adrian John Dunlop^33^, Mahmoud Mamdouh Elhabiby^34^, Hussein Elkholy^35^, Nsidibe Francis Essien^36^, Ghandi Ilias Farah^37^, Marica Ferri^38^, Georgios D Floros^39^, Catherine Friedman^40^, Clara Hidalgo Fuderanan^41^, Gilberto Gerra^17^, Abhishek Ghosh^42^, Maka Gogia^43^, Ilias A. Grammatikopoulos^44^, Paolo Grandinetti^45^, Amira Guirguis^46^, David Gutnisky^47^, Paul Steven Haber^48^, Peyman Hassani-Abharian^49^, Zahra Hooshyari^50^, Islam Ibrahim Mokhtar Ibrahim^34^, Hada Fong-ha Ieong^51^, Regina Nova Indradewi^52^, Shelly Iskandar^53^, Thahir Noorul Isra^54^, Shobhit Jain^55^, Sandi James^56^, Seyyed Mohammad hossein Javadi^57^, Keun Ho Joe^58^, Darius Jokubonis^59^, Acka Tushevska Jovanova^60^, Rama Mohamed Kamal^61^, Alexander Ivanov Kantchelov^62^, Preethy Kathiresan^7^, Gary Katzman^63^, Paul Kawale^64^, Audrey Margaret Kern^65^, Felix Henrique Paim Kessler^66^, Sung-Gon Sue Kim^67^, Ann Marie Kimball^68^, Zeljko Kljucevic^69^, Kristiana Siste Kurniasanti^70^, Roneet Lev^71^, Hae Kook Lee^72^, Aiste Lengvenyte^73^, Shaul Lev-ran^74^, Geni Seseja Mabelya^75^, Mohamed Ali El Mahi^76^, J. Maphisa Maphisa^77^, Icro Maremmani^78^, Laura Masferrer^79^, Omid Massah^80^, Orlagh McCambridge^81^, Garrett Gregory McGovern^82^, Aung Kyi Min^83^, Amir Moghanibashi-Mansourieh^57^, Jazman Mora-Rios^84^, Indika Udaya Kumara Mudalige^85^, Diptadhi Mukherjee^86^, Pejic Munira Munira^87^, Bronwyn Myers^88^, Jayakrishnan Menon T. N.^89^, Venkata Lakshmi Narasimha^90^, Nkemakolam Ndionuka^91^, Ali-Akbar Nejatisafa^92^, Kamran Niaz^17^, Asad Tamizuddin Nizami^93^, Jan H. Nuijens^94^, Laura Orsolini^95^, Vantheara Oum^96^, Adegboyega Adekunle Oyemade^97^, Irena Rojnia Palavra^98^, Sagun Ballav Pant^99^, Joselyn Paredes^100^, Eric Peyron^101^, Randall Alberto Quirós^102^, Rouhollah Qurishi^103^, Noor ul Zaman Rafiq^104^, Ranjini Raghavendra Rao^105^, Woraphat Ratta-apha^106^, Karren-Lee Raymond^107^, Jens Reimer^108^, Eduardo Renaldo^109^, Tara Rezapour^110^, James Roy Robertson^111^, Carlos Roncero^112^, Fazle Roub^113^, Elizabeth Jane Rubenstein^114^, Claudia Ines Rupp^115^, Elizabeth Saenz^17^, Mohammad Salehi^116^, Lampros Samartzis^117^, Laura Beatriz Sarubbo^118^, Nusa Segrec^119^, Bigya Shah^120^, Hongxian Shen^121^, Tomohiro Shirasaka^122^, Steve Shoptaw^123^, Fransiskus Muronga Sintango^124^, Veronica Andrea Sosa^125^, Emilis Subata^126^, Norberto Sztycberg^127^, Fatemeh Taghizadeh^128^, Joseph Brian Tay Wee Teck^129^, Christian Tjagvad^130^, Marta Torrens^131^, Judith Meme Twala^132^, Ramyadarshni Vadivel^133^, Joseph Robert Volpicelli^134^, Jelmer Weijs^135^, Steven Michael Wintoniw^136^, Apisak Wittayanookulluk^137^, Marcin Wojnar^138^, Sadia Yasir^93^, Yimenu Yitayih^139^ and Min Zhao^140^

^1^National Institute for Infectious Diseases, Prof. Dr. Matei Bals- Arena OMT Department, Romania

^2^Faculty of Medicine and Biomedical Sciences, University of Yaoundé 1, Cameroon

^3^Alamal psychiatric hospital, Dubai, United Arab Emirates

^4^Sydney Medical School, University of Sydney, NSW, Australia

^5^Al Ahliyya Amman University, Amman, Jordan

^6^Ministry of Health, Muscat, Oman

^7^Department of Psychiatry and National Drug Dependence Treatment Centre (NDDTC), All India Institute of Medical Sciences (AIIMS), New Delhi, India

^8^State Drug Dependence Treatment Centre, Institute of Mental Health, Pt BDS University of Health Sciences, India

^9^Department of Psychiatry, College of Medicine, University of Ibadan, Nigeria

^10^Addiction Medicine Clinic, Jordan

^11^Drug De-addiction and Treatment Centre, Department of Psychiatry, Postgraduate Institute of Medical Education & Research, Chandigarh, India

^12^Addiction Medicine Clinic, Mila, Algeria

^13^Centre medical Marmottan, France

^14^Réseau de Prévention des Addictions (RESPADD), France

^15^Department of Psychiatry and Behavioural Neurosciences, Michael DeGroote School of Medicine, McMaster University, Hamilton, Ontario, Canada

^16^Geneva University Hospitals, Switzerland

^17^United Nations Office on Drugs and Crime (UNODC), Vienna, Austria

^18^CUNY School of Medicine, New York, United States

^19^Agenzija Sedqa, Malta

^20^Department of Medicine and Surgery, University Milan-Bicocca, Italy

^21^University Psychiatric Hospital Vrapce - Zagreb, Croatia

^22^Department of Addiction Medicine, Haukeland University Hospital, Bergen, Norway

^23^Department of Biochemistry, Melaka Manipal Medical College, Manipal Academy of Higher Education, Manipal-576104, Karnataka, India

^24^Addiction Medicine Clinic, Mexico

^25^Addiction Crisis Solutions, United States

^26^Department of Psychology, Indiana University Purdue University - Indianapolis, United States

^27^SerT Lentini, ASP Siracusa, Italy

^28^Rebirth Charity Society NGO, Tehran, Iran

^29^Serenity Vista Addiction Treatment Center, Panama.

^30^Department of Psychiatry, ESIC Medical College, Hyderabad, Telangana, India

^31^National Institute of Mental Health, Czech Republic

^32^Collaborative Antwerp Psychiatric Research Institute (CAPRI), Antwerp University (UA), Belgium

^33^Drug & Alcohol Clinical Services, Hunter New England Local Health District, Australia

^34^Ain Shams University, Cairo, Egypt

^35^Department of Neurology and Psychiatry, Faculty of Medicine, Ain Shams University, Cairo, Egypt

^36^Centre for Research and Information on Substance Abuse, Nigeria

^37^Addiction Medicine Clinic, Syria

^38^European Monitoring Centre for Drugs and Drug Addiction (EMCDDA), Italy

^39^Department of Psychiatry, Aristotle University of Thessaloniki, Greece

^40^Brown University and Lifespan Health System, Providence, Rhode Island, United States

^41^Fuderanan Mental Health Clinic, Philippines

^42^Drug De-addiction & Treatment Centre, Department of Psychiatry, Postgraduate Institute of Medical Education & Research, Chandigarh, India

^43^Georgian Harm Reduction Network, Georgia

^44^Organization Against Drugs, Primary Care Health Center, Veria, Greece

^45^Addictions Services (Ser.D.), Department of Territorial Services, ASL Teramo, Italy

^46^Swansea University Medical School, Institute of Life Sciences 2, Singleton Campus, SA2 8PP, Wales, United Kingdom

^47^Universidad de Buenos Aires, Argentina

^48^University of Sydney, Australia

^49^Institutes for Cognitive Science Studies (IRICSS), Brain and Cognition Clinic, Tehran, Iran

^50^Tehran University of Medical Sciences, Tehran, Iran

^51^Department of Anesthesiology, Yale University, United States

^52^Drugs Rehabilitation Center, National Narcotics Board of Indonesia, Indonesia

^53^Department of Psychiatry, Universitas Padjadjaran, Bandung, West Java, Indonesia

^54^National Institute of Education, Sri Lanka

^55^Department of Psychiatry, Heritage Institute of Medical Sciences (HIMS), Varanasi, India

^56^Univeristi Malaysia Sabah, Malaysia

^57^Department of Social Work, University of Social Welfare & Rehabilitation Sciences, Tehran, Iran

^58^National Center for Mental Health of Korea, South Korea

^59^Republican Center for Addictive Disorders, Lithuania

^60^Addiction Medicine Clinic, North Macedonia

^61^Naufar Institute, Doha, Qatar

^62^The Kantchelov Clinic, Sofia, Bulgaria

^63^Mount Sinai Medical Center, New York, United States

^64^African Institute for Development Policy, Malawi

^65^Sobriety Centers of New Hampshire, United States

^66^Federal University of Rio Grande do Sul, Brazil

^67^Pusan National University Yangsan Hospital, Department of Neuropsychiatry, Yangsan South Korea

^68^Chatham House, United States

^69^Institute for Public Health of Split-Dalmatia County, Croatia

^70^Faculty of Medicine, Universitas Indonesia-Ciptomangunkusumo Hospital, Indonesia

^71^Scripps Mercy Hospital, San Diego, United States

^72^Department of Psychiatry, The Catholic University of Korea, Seoul, Korea

^73^Faculty of Medicine, Institute of Clinical Medicine, Psychiatric Clinic, Vilnius University, Vilnius, Lithuania

^74^Israel Center on Addiction, Netanya, Israel

^75^Community Health Work, Tanzania

^76^Hayat Center for Treatment and Psycho-social Rehabilitation, Khartoum, Sudan

^77^University of Botswana, Botswana

^78^V.P. Dole, Dual Disorder Unit, Santa Chiara University Hospital, University of Pisa, Italy

^79^CAS Girona, Department of Psychology, University of Girona, Spain

^80^Substance Abuse and Dependence Research Center, University of Social Welfare and Rehabilitation Sciences, Tehran, Iran

^81^Community addiction team, Southern Health and Social Care Trust, Northern Ireland, United Kingdom

^82^Priority Medical Clinic, Dublin, Ireland

^83^Save the Children International Organization, Burma

^84^Dirección de Investigaciones Epidemiológicas y Sociales, Instituto Nacional de Psiquiatría Ramón de la Fuente Muñiz, México

^85^Department of Psychiatry, Faculty of Medicine, Sir John Kotelawala Defence University, Sri Lanka

^86^Centre for Addiction Medicine, NIMHANS, Bangalore, India

^87^Kleopatra Kodric, Irena Nisic, Slovenia

^88^Alcohol Tobacco and Other Drug Research Unit, South African Medical Research Council, South Africa

^89^NIMHANS, Bangalore, India

^90^Centre for Addiction Medicine, Department of Psychiatry, National Institute of Mental Health and Neurosciences, Bengaluru, India

^91^Federal Neuropsychiatric Hospital, Calabar, Nigeria

^92^Department of Psychiatry, Psychosomatic Research Center, Tehran University of Medical Sciences, Tehran, Iran

^93^Institute of Psychiatry, WHO Collaborating Center for Mental Health, Pakistan

^94^Brijder Addiction Care, Zaandam, Netherlands

^95^Department of Clinical Neurosciences/DIMSC, School of Medicine, Polytechnic University of Marche, Ancona, Italy

^96^Koh Kong Provincial Hospital, Cambodia.

^97^Kaiser Permanente, United States

^98^Psychiatric hospital Sveti Ivan, Zagreb, Croatia

^99^Department of Psychiatry and mental health, Institute of Medicine, Tribhuvan University, Nepal

^100^Universidad de El Salvador, El Salvador

^101^AddiPsy, Lyon, France

^102^Addiction Medicine Clinic, Costa Rica

^103^Novadic-Kentron Addiction Care Network, Vught, Netherlands

^104^Phoenix Foundation for Research and Development, Pakistan

^105^Barwon Health, Geelong, Australia

^106^Faculty of Medicine Siriraj Hospital, Mahidol University, Thailand

^107^University of the Sunshine Coast (USC), Queensland, Australia

^108^Center for Interdisciplinary Addiction Research, University Medical Center Hamburg-Eppendorf, Hamburg, Germany

^109^Drugs Rehabilitation Center, National Narcotics Board of Indonesia

^110^Department of Cognitive Psychology, Institute for Cognitive Science Studies, Tehran, Iran

^111^Usher Institute, University of Edinburgh, United Kingdom

^112^Psychiatry Service, University of Salamanca Health Care Complex, Salamanca, Spain

^113^PGIMER, Chandigarh, India

^114^Street Health Centre, Canada

^115^Department of Psychiatry, Psychotherapy, and Psychosomatics, Medical University Innsbruck Austria

^116^Department of Neurosciences and Addiction Studies, School of Advanced Technologies in Medicine, Tehran University of Medical Sciences, Tehran, Iran

^117^Medical School, University of Cyprus, Cyprus

^118^Clínica Psiquiátrica de la Facultad de Medicina, Uruguay

^119^Center for Treatment of Drug addiction, University Psychiatric Clinic, Ljubljana, Slovenia

^120^Department of Psychiatry, Patan Academy of Health Sciences, School of Medicine, Lagankhel, Nepal

^121^Department of Psychiatry, Second Xiangya Hospital, Central South University, China

^122^Department of Psychiatry, Teine Keijinkai Medical Center, Japan

^123^David Geffen School of Medicine at UCLA, Department of Family Medicine, United States

^124^Health Professions Councils of Namibia, Namibia

^125^Addiction Medicine Clinic, Uruguay

^126^Republican Center for Addictive Disorders, Lithuania

^127^Asociasion Programa Andres Argentina, Argentina

^128^Mazandaran University of Medical Sciences, Mazandaran, Iran

^129^MRC/CSO SPHSU, University of Glasgow, United Kingdom

^130^Gladsaxe Substance Use Disorder Treatment Centre, Denmark

^131^Institut de Neuropsiquiatria i Addiccions, IMIM-Hospital del Mar, Medical Research Barcelona, Spain

^132^NACADA, Kenya

^133^Waikato District Health Board (WDHB) Hamilton, New Zealand

^134^Institute of Addiction Medicine, United States

^135^Jellinek, Amsterdam, Netherlands

^136^Addictions Foundation of Manitoba, Canada

^137^Thanyarak Chiangmai Hospital, Thailand

^138^Medical University of Warsaw, Warsaw, Poland

^139^Jimma University, Ethiopia

^140^Shanghai Mental Health Center, Shanghai Jiao Tong University School of Medicine, China

## Conflict of Interest

The authors declare that the research was conducted in the absence of any commercial or financial relationships that could be construed as a potential conflict of interest.

## Publisher's Note

All claims expressed in this article are solely those of the authors and do not necessarily represent those of their affiliated organizations, or those of the publisher, the editors and the reviewers. Any product that may be evaluated in this article, or claim that may be made by its manufacturer, is not guaranteed or endorsed by the publisher.

## Footnotes

^1^ https://www.deadiversion.usdoj.gov/GDP/(DEA-DC-028)(DEA084)_Hospital_Clinic_Registration_Exception_(final).pdf
