# Supplementary material for: A Global Survey on Changes in the Supply, Price, and Use of Illicit Drugs and Alcohol, and Related Complications During the 2020 COVID-19 Pandemic
Source: Front Psychiatry. 2021 Aug 6;12:646206. doi: 10.3389/fpsyt.2021.646206 (PMC8377291; doi:10.3389/fpsyt.2021.646206)
Supplement: Supplementary file 1 [file Data_Sheet_1.docx]

**Supplementary materials**

**Supplementary Methods:**

**Supplementary Method 1. Respondents' global distribution.** Overall, 177 respondents from 77 countries participated in the survey. The countries that provided information for this survey consisted of Afghanistan, Algeria, Argentina, Australia, Austria, Belgium, Bolivia, Botswana, Brazil, Bulgaria, Burma, Cambodia, Cameroon, Canada, China, Costa Rica, Croatia, Cyprus, Czech Republic, Denmark, Egypt, El Salvador, Ethiopia, France, Georgia, Germany, Greece, India, Indonesia, Iran, Iraq, Ireland, Israel, Italy, Japan, Jordan, Kenya, Lithuania, Macau, Malawi, Malaysia, Malta, Mexico, Namibia, Nepal, Netherlands, New Zealand, Nigeria, North Macedonia, Norway, Oman, Pakistan, Palestine, Panama, Philippines, Poland, Qatar, Romania, Slovenia, Somalia, South Africa, South Korea, Spain, Sri Lanka, Sudan, Sweden, Switzerland, Syria, Tanzania, Thailand, Turkey, Ukraine, United Arab Emirates, United Kingdom, United States, Uruguay, Zimbabwe.

**Supplementary Method 2. Survey questions related to the situational assessment.** The situational assessment section composed of the following questions: “Which of these substances went through changes in terms of usage pattern in your country?” (Increased/ decreased/ no changes), “Which of these substances went through changes in terms of price in your country?” (Increased/ decreased/ no changes), “Which of these substances went through changes in terms of supply in your country?” (Increased/ decreased/ no changes). The questioned substances included alcoholic beverages, cannabis (i.e. marijuana and synthetic cannabinoids such as spice, K2, etc.), opiates (i.e. opium, heroin, opium residue, etc.), amphetamine type stimulants (i.e. amphetamine, methamphetamine, MDMA, etc.), cocaine (including crack cocaine), sedative and hypnotics (i.e. Benzodiazepines, Barbiturates, etc.), and prescription opioids (e.g., Oxycodone, Oxycontin, Tramadol, Diphenoxylate, etc.). Other questions were “Have morbidities or mortalities increased in people with alcohol use disorders (AUDs) during the period of this pandemic?” (Severe, slight, no change in morbidities or mortalities), “Have morbidities or mortalities increased in people with SUDs during the period of this pandemic?” (Severe, slight, no change in morbidities or mortalities), “Have fatal and non-fatal overdose episodes changed among people with SUDs during the period of this pandemic?” (Severe, slight, no change in overdoses), “Have there been any changes in risky behaviors among people with SUDs during the period of this pandemic?” (risky behaviors consist of increased/switch to injection, sharing drug use equipment, needle and syringe sharing, and risky sexual behaviors), and “In general, how serious do you think people with SUDs in your country are affected by the COVID-19 outbreak?” (rate the severity on a 0-10 Likert scale).

**Supplementary Results:**

**Drug use changes**

Over 63% (n=49) of the countries reported that alcohol use increased, whereas 25% (n=19) and 8% (n=6) reported a decrement and no change in alcohol use pattern, respectively. Approximately 4% (n=3) responded that they do not know or are unwilling to answer this question. Approximately 42% of the countries (n=32) reported that cannabis use increased, whereas 26 % (n=20) and 22% (n=17) reported a decrease and no change in cannabis use pattern, respectively. Approximately 10% (n=8) responded that they do not know or are unwilling to answer this question. Approximately 18% (n=14) of the reported that opiates use increased, whereas 31% (n=24) and 20% (n=16) reported a decrement and no change in opiates use pattern, respectively. Approximately 30% (n=23) responded that they do not know or are unwilling to answer this question. Approximately 18% (n=14) of the countries reported that amphetamines use increased, whereas 29% (n=22) and 20% (n=15) reported a decrement and no change in amphetamines use pattern, respectively. Approximately 33% (n=26) responded that they do not know or are unwilling to answer this question. Approximately 14% (n=10) of the countries reported that cocaine use increased, whereas 29% (n=23) and 19% (n=15) reported a decrement and no change in cocaine use pattern, respectively 38% (n=29) responded that they do not know or are unwilling to answer this question. Approximately 64% (n=50) of the reported that sedatives and hypnotics use increased, whereas 6% (n=5) and 11% (n=9) reported a decrement and no change in sedatives and hypnotics use pattern, respectively. Approximately 18% (n=14) responded that they do not know or are unwilling to answer this question. Approximately 41% (n=32) of the countries reported that prescription opioids use was increased, whereas 11% (n=8) and 21% (n=16) reported a decrement and no change in prescription opioids use pattern, respectively. Approximately 27% (n=21) responded that they do not know or are unwilling to answer this question.

**Drug supply changes**

Over 31% (n=24) of the countries reported that the alcohol supply increased, whereas 34% (n=26) and 28% (n=21) reported a decrement and no change in the alcohol supply pattern, respectively. Approximately 7% (n=6) responded that they do not know or are unwilling to answer this question. 20% (n=15) of the countries reported that the cannabis supply increased, whereas 37 % (n=29) and 24% (n=18) reported a decrement and no change in the cannabis supply pattern, respectively. Approximately 19% (n=15) responded that they do not know or are unwilling to answer this question. Only 8% (n=6) of the countries reported that opiates supply increased, whereas 41% (n=31) and 18% (n=14) reported a decrement and no change in opiates supply pattern, respectively. Approximately 33% (n=26) responded that they do not know or are unwilling to answer this question. Only 9% (n=7) of the countries reported that amphetamines supply increased, whereas 38% (n=29) and 18% (n=14) reported a decrement and no change in amphetamines supply pattern, respectively. Approximately 35% (n=27) responded that they do not know or are unwilling to answer this question. Only 9% (n=7) of the countries reported that the cocaine supply was increased, whereas 34% (n=24) and 18% (n=14) reported a decrement and no change in cocaine supply pattern, respectively. Approximately 39% (n=30) responded that they do not know or are unwilling to answer this question.

**Drug price changes**

Over 29% (n=23) of the countries reported that alcohol price increased, whereas 4% (n=3) and 54% (n=42) reported a decrement and no change in alcohol price, respectively. Approximately 13% (n=10) responded that they do not know or are unwilling to answer this question. 39% (n=30) of the countries reported that cannabis price increased, whereas 3% (n=2) and 30% (n=23) reported a decrease and no change in cannabis price, respectively. Approximately 28% (n=22) responded that they do not know or are unwilling to answer this question. 37% (n=29) of the countries reported that opiates price increased, whereas 2% (n=2) and 18% (n=14) reported a decrement and no change in opiates price, respectively. Approximately 43% (n=33) responded that they do not know or are unwilling to answer this question. 28% (n=21) of the countries reported that amphetamines price increased, whereas 0% (n=0) and 23% (n=18) reported a decrement and no change in amphetamines price, respectively. Approximately 49% (n=37) responded that they do not know or are unwilling to answer this question. Approximately 43% (n=33) of the countries reported that cocaine price increased, whereas 3% (n=2) and 39% (n=30) reported a decrease and no change in cocaine price, respectively. Approximately 15% (n=12) responded that they do not know or are unwilling to answer this question.

**Morbidities, mortalities and overdose rate changes**

41% (n=72) of respondents reported that morbidities and mortalities have increased among people with AUDs during this pandemic, while 4% (n=7) and 19% (n=34) reported a decrement and no change regarding this matter, respectively. 36% (n=64) responded that they do not know or are unwilling to answer this question. Approximately 38% (n=68) of the reported that morbidities and mortalities have increased among people with SUD during this pandemic, while 4% (n=7) and 19% (n=34) reported a decrement and no change regarding this matter, respectively. Approximately 38% (n=68) responded that they do not know or are unwilling to answer this question. Approximately 20% (n=35) of the respondents reported that fatal and non-fatal overdoses have increased among people with SUD, while 8% (n=14) and 30% (n=53) reported a decrement and no change in overdoses, respectively. Approximately 42% (n=75) responded that they do not know or are unwilling to answer this question.

**Supplementary Figure:**


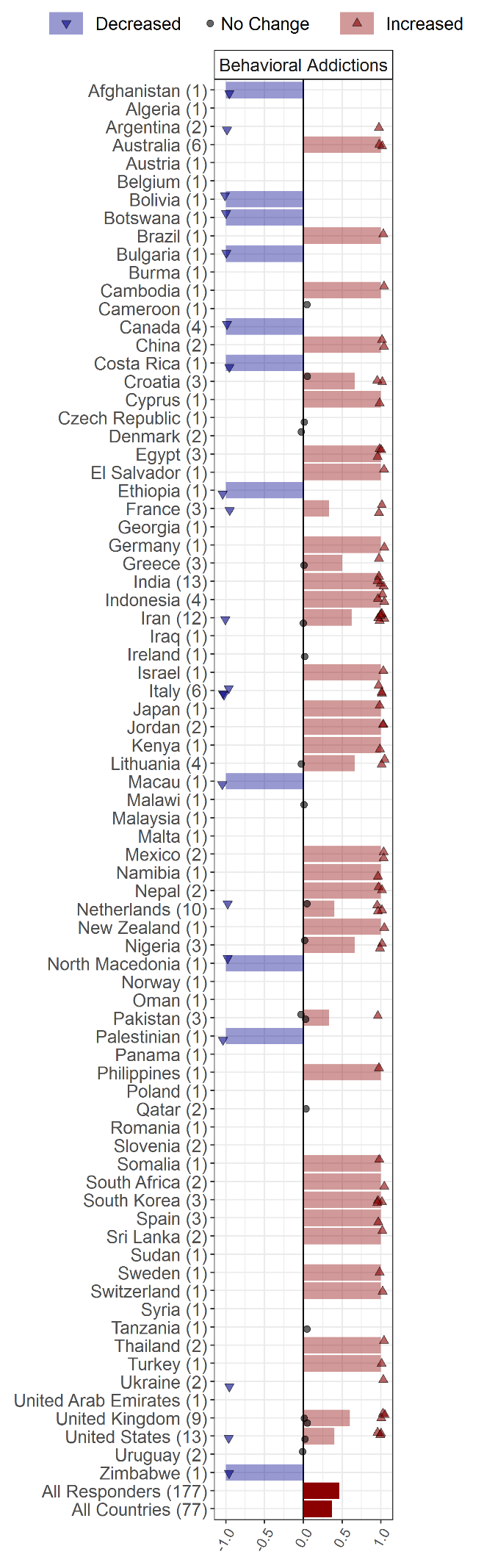


**Supplementary Figure 1. Changes in behavioral addictions including gambling and gaming during the COVID-19 pandemic, reported by 177 respondents from 77 countries around the world.** Respondents were asked to report changes in behavioral addiction rates in their countries through the following options: *Increased, Decreased, No change, I do not know.* Countries’ names are sorted in alphabetical order, and the number of each countries’ respondents is mentioned in front of the names. Each response is indicated as a single dot for *no change* or up and down triangles for *increased* and *decreased* answers, respectively, with a minor jitter for better visualization. The reported answers are represented as -1 for *decreased*, 1 for *increased,* and 0 for *no change; I do not know* answers are not shown in the figure. The mean of all responses, regardless of their originated countries and without considering those who didn’t know the answer, alongside the average answers of all countries, regardless of the number of respondents in each country, are addressed in the last two rows below the countries’ names. The rates of behavioral addictions in the countries and respondents that responded to this question have been increased by 40% and 50%, respectively. Approximately 86% (n=66) of the countries reported that behavioral addictions rates had increased, whereas 14% (n=11) of the countries reported that behavioral addictions rates had decreased in their countries during the COVID-19 pandemic.

**Supplementary Tables:**

**Supplementary Table 1.** Information related to risky behaviors including injection, sharing drug use equipment, needle and syringe sharing, and risky sexual behaviors among respondents and countries.

|  |  |  |  |  |  |  |  |  |  |  |  |  |  |  |  |  |  |
| --- | --- | --- | --- | --- | --- | --- | --- | --- | --- | --- | --- | --- | --- | --- | --- | --- | --- |
|  | Respondents (177) | | | | | | | |  | Countries (77) | | | | | | | |
|  | No | |  | Yes | |  | Others | |  | No | |  | Yes | |  | Others | |
|  | N | % |  | N | % |  | N | % |  | N | % |  | N | % |  | N | % |
| Injection | **58** | **33%** |  | 29 | 16% |  | 90 | 51% |  | **28** | **36%** |  | 10 | 13% |  | 39 | 50% |
| Shared Drug Use Equipment | **44** | **25%** |  | 41 | 23% |  | 92 | 52% |  | **20** | **26%** |  | 18 | 24% |  | 39 | 50% |
| Needle Sharing | **43** | **24%** |  | 38 | 21% |  | 96 | 54% |  | **21** | **27%** |  | 16 | 21% |  | 40 | 52% |
| Risky Sexual Behaviors | 39 | 22% |  | **41** | **23%** |  | 97 | 55% |  | **20** | **25%** |  | 19 | 25% |  | 38 | 49% |
|  |  |  |  |  |  |  |  |  |  |  |  |  |  |  |  |  |  |

The mean number and percentage of *Yes, No and Others* responses, regardless of their originated countries and the average answers of all countries, regardless of the number of respondents in each country. *‘Others’* indicate responses that involved respondents’ lack of information or reluctance for responding to the relevant question.
